# A new immunochemical strategy for triple-negative breast cancer therapy

**DOI:** 10.1038/s41598-021-94230-4

**Published:** 2021-07-21

**Authors:** Chih-Wei Lin, Tianqing Zheng, Geramie Grande, Alex R. Nanna, Christoph Rader, Richard A. Lerner

**Affiliations:** 1 Department of Chemistry, The Scripps Research Institute, La Jolla, CA 92037 USA; 2 Department of Immunology and Microbiology, The Scripps Research Institute, Jupiter, FL 33458 USA

**Keywords:** Biotechnology, Cancer, Molecular biology, Chemical biology

## Abstract

Triple-negative breast cancer (TNBC) is a highly diverse group of malignant neoplasms which tend to have poor outcomes, and the development of new targets and strategies to treat these cancers is sorely needed. Antibody–drug conjugate (ADC) therapy has been shown to be a promising targeted therapy for treating many cancers, but has only rarely been tried in patients with TNBC. A major reason the efficacy of ADC therapy in the setting of TNBC has not been more fully investigated is the lack of appropriate target molecules. In this work we were able to identify an effective TNBC target for use in immunotherapy. We were guided by our previous observation that in some breast cancer patients the protein tropomyosin receptor kinase B cell surface protein (TrkB) had become immunogenic, suggesting that it was somehow sufficiently chemically different enough (presumably by mutation) to escaped immune tolerance. We postulated that this difference might well offer a means for selective targeting by antibodies. We engineered site-specific ADCs using a dual variable domain (DVD) format which combines anti-TrkB antibody with the h38C2 catalytic antibody. This format enables rapid, one-step, and homogeneous conjugation of β-lactam-derivatized drugs. Following conjugation to β-lactam-derivatized monomethyl auristatin F, the TrkB-targeting DVD-ADCs showed potency against multiple breast cancer cell lines, including TNBC cell lines. In addition, our isolation of antibody that specifically recognized the breast cancer-associated mutant form of TrkB, but not the wild type TrkB, indicates the possibility of further refining the selectivity of anti-TrkB DVD-ADCs, which should enhance their therapeutic index. These results confirmed our supposition that TrkB is a potential target for immunotherapy for TNBC, as well as for other cancers with mutated cell surface proteins.

## Introduction

Prior to 1986 there were no approved therapeutic antibodies in the clinic. Since that time there has been an explosion in the number of Food and Drug Administration (FDA) approved antibodies. Basically, these antibodies fall into two broad classes. In the first class, antibodies are simply used as scavenger molecules to sequester effectors, such as TNF and complement, that are over-produced in diseases such as rheumatoid arthritis and ulcerative colitis. The second class of antibodies is used to perturb the vast network of immunological regulatory molecules, such as various lymphokines and cytokines, and more recently, the programed death receptor and its ligand, PD1 and PDL1, respectively^1–3^. Upon viewing this landscape, one might ask why, in the setting of cancer, is so much attention paid to regulators of the immune response, rather than to the tumor itself. The answer to this question is that true tumor-specific antigens are rare.

Nothing illustrates this problem better than breast cancer. Breast cancer is the most common malignancy in women, with approximately 266,000 new cases and 41,000 deaths reported in the United States during 2018 alone^4^. Breast cancer is the second leading cause of cancer death in women, after lung cancer, and is a heterogeneous disease that consists of multiple subtypes that respond distinctly to different therapeutic regimens^5,6^. Although some targeted therapies for breast cancer have recently been approved for use, there remain few therapeutic options for metastatic triple-negative breast cancer (TNBC) patients. TNBC is defined by the lack of expression of the estrogen receptor (ER), the progesterone receptor (PR), and the lack of expression or amplification of the human epidermal growth factor receptor 2 (HER2)^7^. TNBC accounts for approximately 15–25% of invasive breast cancers. It is associated with aggressive tumor biology and a poor prognosis. TNBC most commonly metastasizes to the brain, bone and visceral organs such as lung, liver^8–11^. Therapies targeting this virulent form of breast cancer are sorely needed.

It has recently been shown that somatic mutations in growth factor receptors affect cancer growth and treatment outcomes^12,13^. For example, studies have shown that mutations in the NTRK gene, which encodes for a family of growth factor receptors (protein tropomyosin receptor kinases, Trk) have been found in some cancer patients. Initially, Trk receptors were thought to only affect neuron survival and differentiation through several signal cascades. However, later it was discovered that mutated or fusion forms of one of the Trk proteins (TrkB) can lead cancer cells to grow, and may adversely affect the efficacy of drug treatment. This knowledge has been used to develop novel targets for cancer therapy across multiple tumor types^14,15^. We selected TrkB as the target in the experiments reported here because of our earlier finding that some patients with breast cancer make antibodies to TrkB. We previously reported the identification and characterization of anti-TrkB monoclonal antibodies (mAbs)^16^ derived from such patients. These human antibodies were of high affinity to TrkB, and suggested that TrkB would be a good therapeutic target if selective antibodies could be generated. However, two issues needed to be addressed. First, some of the antibodies were agonists, with the potential to drive tumor growth. Second, in terms of immunogenicity these proteins expressed on cancer cells may be only subtly different from their normal counterparts, and thus theoretically, it might be difficult to generate selective antibodies to them. But most important, the fact that some patients make antibodies to these proteins suggests that in the cancer setting they can have a differential immunogenicity, likely as the result of somatic mutation, and this differential immunogenicity could be exploited for the generation of tumor-specific therapies. The central idea in the current study is that modern immunochemical technology, especially combinatorial antibody libraries and newer selection systems, are sufficiently powerful to generate highly selective antibodies even to only slightly modified mutant proteins.

Once we selected TrkB as the target, the next question was, what kind of therapeutic modality should we construct. Recently, antibody–drug conjugates (ADCs) have emerged as a powerful class of therapeutics in the targeted treatment of cancer. To date, three ADCs have received FDA approval for treating breast cancer. Two of these ADCs target HER2, which is overexpressed in only 15 to 25% of all breast cancer patients. One ADC targets trophoblast cell surface antigen 2 (Trop-2). The majority of methods for generating ADCs use random conjugation to cysteine (Cys) or lysine (Lys) residues. However, these methods result in a heterogeneous mixture of ADCs. Heterogeneous ADCs usually contain 0–8 drug molecules and have undesired pharmacological properties^17^. For these reasons, several site-specific conjugation technologies have been developed to prepare more homogeneous ADCs^18–20^. Producing homogeneous ADCs usually relies on mutations or inefficient conjugation chemistries. Recently, the development of site-specific ADCs using the highly reactive, naturally-embedded lysine of a catalytic antibody integrated in a dual variable domain (DVD) format was reported^21,22^. This method does not rely on mutation, and drug conjugation at neutral pH is fast, clean, and efficient. The upper variable domain is a targeting antibody that locks onto a cancer cell, while the lower variable domain is a catalytic antibody that carries the drug. Humanized 38C2 mAb (h38C2) was shown to be a suitable catalytic antibody for this method. MAb h38C2 has a reactive heavy chain variable region lysine (Lys99) deeply buried in the hydrophobic base of the antibody binding pocket, and thus, is not protonated at physiological pH. This nucleophilic lysine catalyzes aldol and retro-aldol reactions using an enamine mechanism^23,24^. As such, catalytic antibody h38C2 can be selectively conjugated to 1,3-diketone or β-lactam derivatives, and provides highly selective covalent attachment of the targeting modules or payload^25–31^. It has been reported that DVDs (~ 200 kDa) have similar pharmacokinetic and tissue penetration properties as IgGs (~ 150 kDa) and retain a functional Fc domain for FcRn binding. This format has also been applied clinically, with two DVDs currently in phase II clinical trials^32,33^. For these reasons, we chose this DVD format as our therapeutic modality.

In the studies reported here, we developed a one-step assembled, TrkB-targeting DVD-ADC using β-lactam-derivatized tubulin inhibitor monomethyl auristatin F (MMAF). We showed its in vitro efficacy against a range of breast cancer cell lines, including three triple negative cell lines. We demonstrated for the first time that TrkB is a candidate target for ADCs, an advance which holds significant promise for the treatment of TNBCs. In addition, we also developed a general method for generating antibodies against mutated growth factor receptors and other proteins on cancer cell surfaces, thereby extending the list of potential targets for cancer immunotherapy.

## Results

### Determination of TrkB expression in breast cancer cell lines

**Figure 1:**
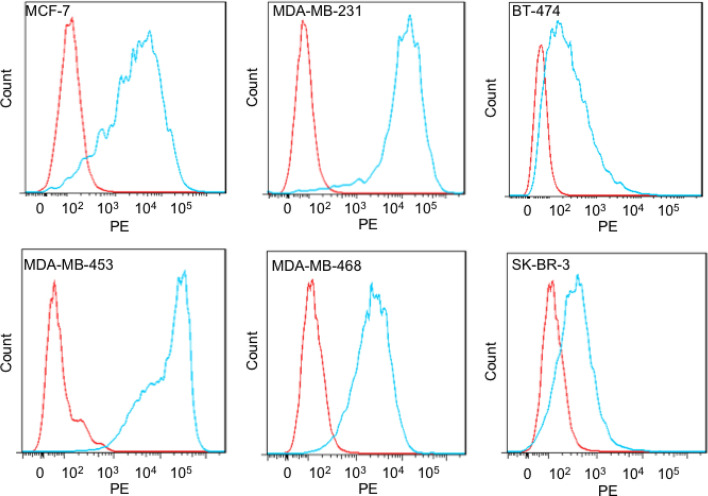
Flow cytometric analysis of TrkB expression in breast cancer cell lines. Flow cytometric analysis of six breast cancer cell lines using anti-TrkB mAb 641 (blue) or an isotype control mAb (red) detected by a secondary anti-human-PE antibody.

The anti-TrkB mAb has been previously described and shown to have anti-tumor effects on breast cancer cells^16^. In order to understand the expression level of TrkB in breast cancer cells, we analyzed the expression of TrkB using mAb 641 in six human breast cancer cell lines using flow cytometry. All cell lines showed TrkB expression at various levels (Fig. 1). TNBC cell lines MDA-MB-231, MDA-MB-453, and MDA-MB-468 revealed the strongest staining.

### Anti-TrkB mAb internalization

To assess the cellular uptake of anti-TrkB mAb 641, the antibody was conjugated to a pH sensor dye (pHAb dye). pHAb dye shows low fluorescent at neutral pH but becomes highly fluorescent at acidic pH. When the anti-TrkB mAb conjugated to pHAb dye binds to its antigen on the cancer cell membrane, the antibody-dye-antigen complex is not fluorescent, but upon internalization and trafficking into endosomal and lysosomal vesicles, the pH drops, and the dye becomes fluorescent. MDA-MB-468 cells were incubated with anti-TrkB mAb 641 conjugated to pHAb for 1–8 h at 37°C. The fluorescence was measured by flow cytometry and showed an accumulation over 4 h. Another TNBC cell line, MDA-MB-231, gave similar results (Supplementary, Fig. S1A and B), indicating that after binding to TrkB on the cell surface the antibody is internalized.

### Construction of DVD format using anti-TrkB and h38C2 antibodies

**Figure 2:**
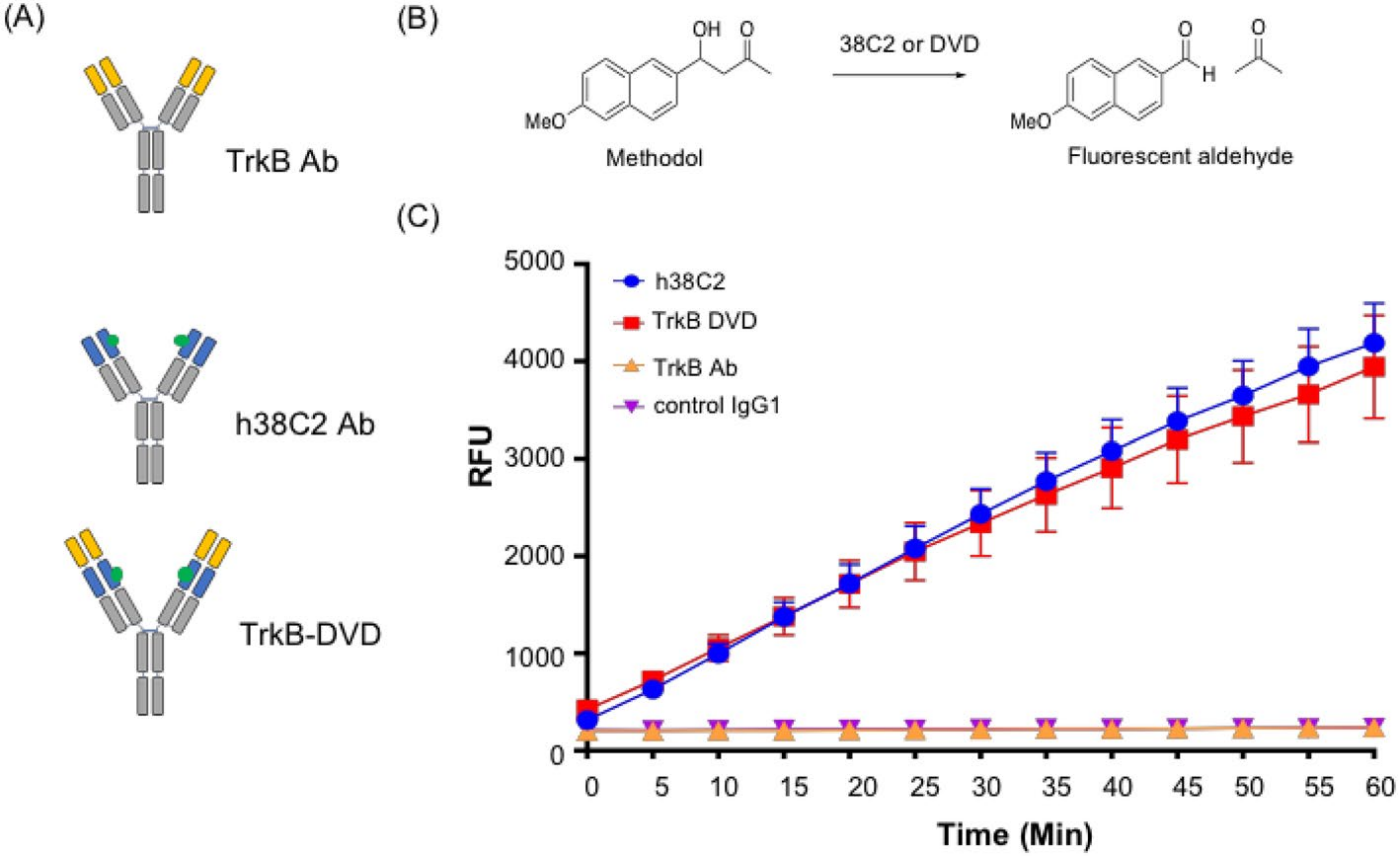
Construction of a TrkB-targeting DVD. (**A**) The DVD is composed of the variable domains of anti-TrkB mAb 641 (yellow), catalytic antibody h38C2 (blue) with its reactive Lys99 residue (green circle), and constant domains (gray). A short hexapeptide linker (ASTKGP) was used between the variable domains. (**B**) Structure of methodol and its parent fluorescent aldehyde and acetone following a retro-aldol reaction catalyzed by h38C2 or DVD. (**C**) The catalytic retro-aldol activity was measured using methodol as a substrate. The signal is reported in relative fluorescent units (RFU; mean ± SD of triplicates). Anti-TrkB and an isotype control IgG1 were used as negative controls. Abbreviation: dual variable domain (DVD), relative fluorescence units (RFU).

We engineered a TrkB-targeting DVD by combining the variable domains of anti-TrkB mAb 641 and catalytic antibody h38C2 to enable tumor targeting and drug conjugation. The constructed DVD is a h38C2 IgG1 with the variable domains of anti-TrkB mAb 641 incorporated at the N-termini of heavy and light chains (Fig. 2A). A short hexapeptide linker (ASTKGP) was used between the two variable domains. The DVD expression cassettes were cloned into the mammalian expression vector pcDNA3.1 using *BamH* and *PmeI* restriction enzyme sites (Supplementary, Fig. S2A). Thus, there are two TrkB binding sites and two drug attachment sites within one DVD molecule. To ensure retention of the h38C2 Lys99 reactivity in the DVD format, we used an assay directly assessing its catalytic activity through the conversion of methodol to its parent fluorescent naphal aldehyde (Fig. 2B) via a retro-aldol reaction. The formation of the fluorescent aldehyde was detected by measuring the emission at 452 nm. The TrkB-targeting DVD was found to retain the catalytic activity of control h38C2 IgG1. As expected, the anti-TrkB IgG1, which has no reactive Lys residue, had no catalytic activity with methodol (Fig. 2C). The TrkB-targeting DVD was purified (Supplementary, Fig. S2B) and shown to have retained its ability to bind MDA-MB-231 breast cancer cells (Supplementary, Fig. S2C).

### Generation of anti-TrkB DVD-ADC

**Figure 3:**
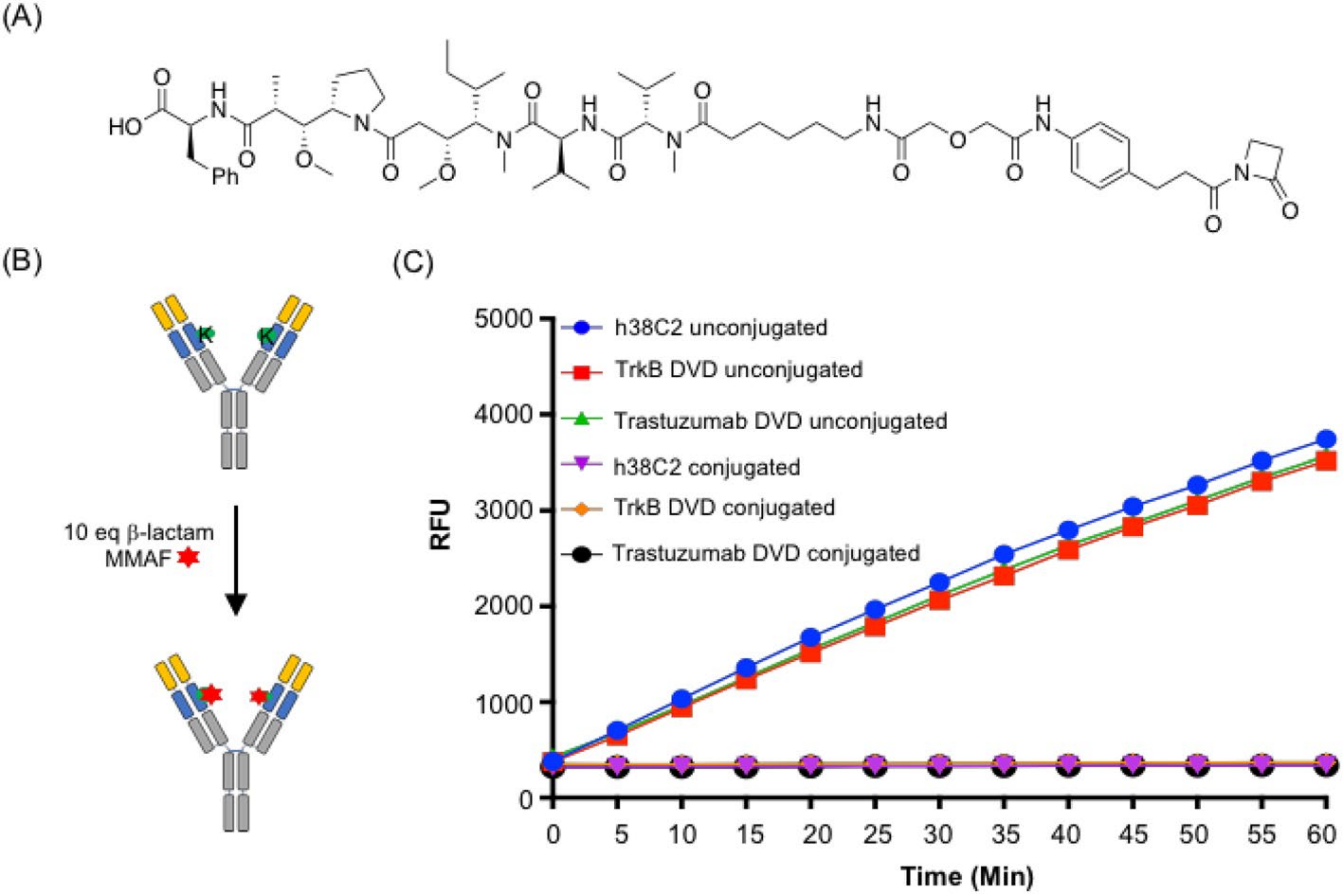
Generation of anti-TrkB DVD-ADCs (**A**) A β-lactam functionalized MMAF compound with a non-cleavable linker was used for ADC assembly. (**B**) The DVD-ADC was prepared by incubating the anti-TrkB DVD with 10 eq of β-lactam MMAF (red star) in PBS for 4 h at room temperature. The reactive Lys99 residue (K) is shown in green. (**C**) As Lys99 reacts with the β-lactam moiety to form an amide bond, DVD is no longer catalytically active, thus confirming complete conjugation. The signal is reported in relative fluorescent units (RFU; mean ± SD of triplicates). Abbreviation: dual variable domain (DVD), relative fluorescence units (RFU).

A β-lactam functionalized monomethyl auristatin F (MMAF) compound with a non-cleavable linker was used to assemble the DVD-ADC (Fig. 3A). MMAF with a non-cleavable linker has been used to construct potent ADCs against a variety of targets. The purified TrkB-targeting DVD was incubated with ten equivalents (10 eq) of β-lactam MMAF (5 eq with respect to each Lys99 residue) in PBS for 4 h at room temperature (Fig. 3B). As the Lys99 residue reacts with the β-lactam moiety to form an amide bond, it is no longer catalytically active. Thus, the complete loss of catalytic activity indicated that conjugation was complete (Fig. 3C). These data show the successful assembly of the anti-TrkB DVD-ADC.

### In vitro cytotoxicity assays

**Figure 4:**
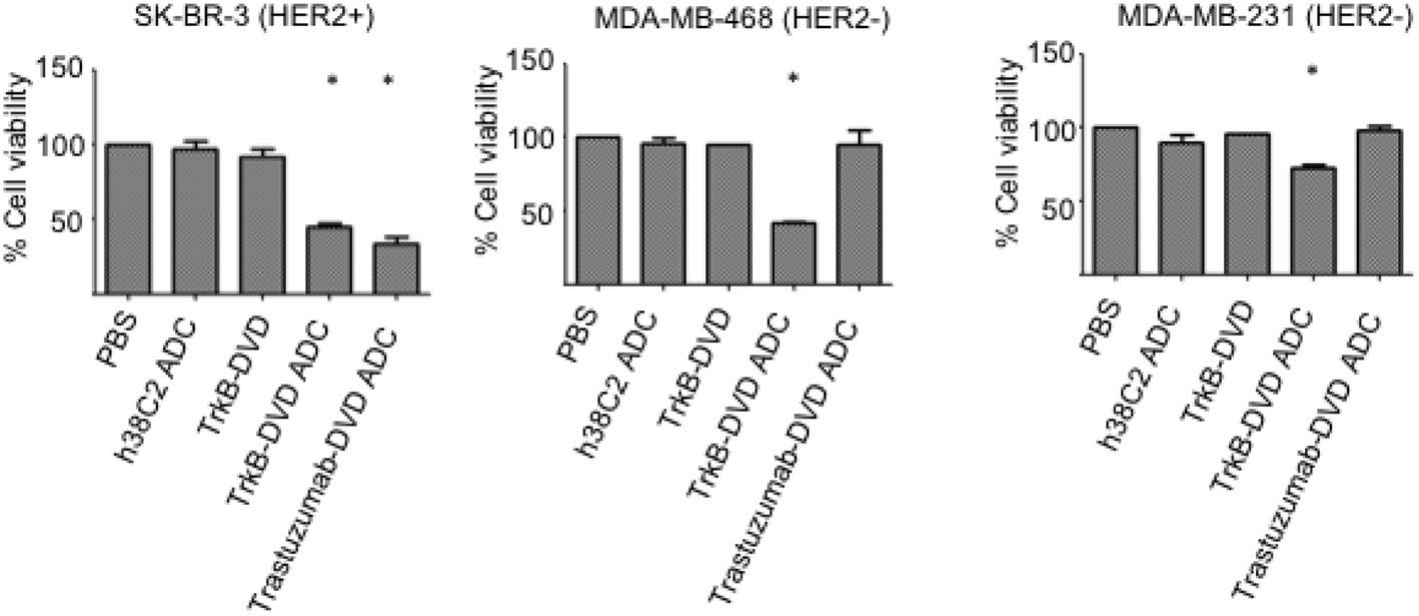
Effect of anti-TrkB DVD ADC on breast cancer cell lines. Cytotoxicity of anti-TrkB DVD-ADC following incubation with HER2+ breast cancer cell line SK-BR-3, and HER2− breast cancer cell lines MDA-MB-231 and MDA-MB-468 for 72 h at 37 °C. Unconjugated anti-TrkB DVD and MMAF-conjugated trastuzumab-based DVD-ADC and h38C2 IgG1-ADC served as controls. Breast cancer cell lines (5 × 10^3^ cells/well) were seeded in 96-well plates and incubated with 100 nM of the various reagents. Cell viability was measured using the CellTiter 96 AQueous One Solution Cell Proliferation Assay kit following the manufacturer’s protocol. Vehicle-treated cells were set to 100% viability. All experiments were performed in triplicate, error bars indicate standard deviation. A t test was used to compare the treatment groups. All statistical evaluations of data were performed using GraphPad Prism 8 software. Statistical significance was achieved at a p value ≤ 0.05. (*, *p* ≤ 0.05).

Given the finding of TrkB antibody internalization following binding, we evaluated the functional activity of the TrkB-targeting DVD-ADC in vitro with respect to mediating breast cancer cell death. We used a previously described trastuzumab-based HER2-targeting DVD-ADC as an internal control to validate the assay. The non-targeting h38C2 IgG1-ADC was used as a negative control. The unconjugated anti-TrkB DVD was also included as a control. The cytotoxic activity of all four reagents was tested against SKBR3, MDA-MB-468 and MDA-MB-231 breast cancer cell lines. In this assay, the breast cancer cells were incubated in growth medium with 100 nM of each of the four reagents for 72 h. As shown in Fig. 4, the anti-TrkB DVD-ADC was cytotoxic for all of the breast cancer cell lines. The greatest potency was observed with MDA-MB-468 cells. In addition, treatment with both anti-TrkB and anti-HER2 DVD-ADCs caused cytotoxicity in the HER2+ SKBR3 cell line. Notably, the anti-TrkB DVD-ADC was cytotoxic for two HER2− breast cancer cell lines (MDA-MB-468 and MDA-MB-231), that were not affected by the anti-HER2 DVD-ADC. These results validate the therapeutic utility of targeting TrkB with ADCs, particularly in HER2− breast cancers, including TNBCs that express TrkB.

### A cell–cell interaction format for selection of antibodies against mutant TrkB

**Figure 5:**
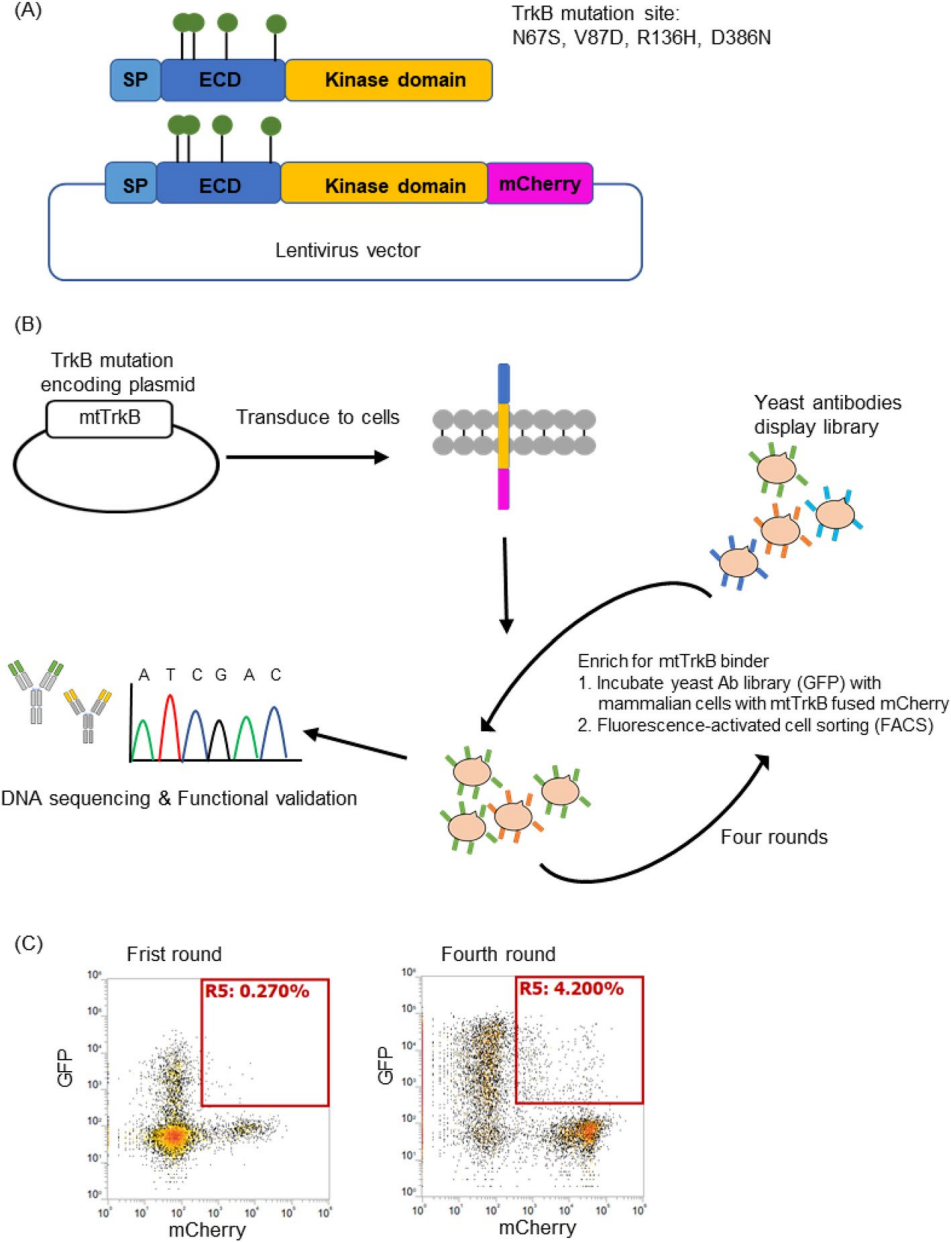
Schematic representation of the yeast display screening against mutated TrkB. (**A**) Schematic representation of the lentivirus expression plasmid encoding the mutated TrkB (mtTrkB) is shown. The four mutated TrkB site was located on extracellular domain (ECD) of TrkB. SP, signal peptide. (**B**) Yeast display was applied to select the four mutated amino acids of TrkB. A mtTrkB plasmid, which consists single or four mutated sites was created. The mtTrkB lentivirus plasmids were produced and transduce to 293 T to generate the overexpressed mtTrkB stable cell lines. The yeast surface display library was then subjected to selection for mtTrkB binding. Different variants antibodies were displayed on yeast cells. We used fluorescence-activated cell sorting (FACS) to enrich yeast cells that were able to interact with mtTrkB cell line. The post-selection pool was then expanded and subjected to another round. Four rounds of selection were performed. Variants that were able to bind to the mtTrkB cell lines would enrich in occurrence frequency throughout the screening process. The post-selection yeast antibodies libraries were sequenced and further expressed to confirm the cell binding of mtTrkB cell lines. (**C**) Yeast library was enriched by round 4 against mutated TrkB cell line.

Since we now knew that our DVD-ADC construct could be used to target TrkB on tumor cells, our attention turned to the important issue of selectivity. Because of the fact that patients with breast cancer made antibody to TrkB, we assumed that in the cancer setting this was the result of altered immunogenicity in this protein, probably because of mutation. Thus, selective recognition of these mutants could inure to the desired tumor-specific reactivity. Furthermore, we believed that our newest selection methodology for combinatorial antibody libraries could yield antibodies selected for the mutant form. The TrkB residues that are mutated in cancers were determined using cBioPorta, which contains multidimensional cancer genomics and clinical data from The Cancer Genome Atlas (TCGA). Their analysis shows that NTRK2 has a somatic mutation frequency of 0.4% in cancer patients. Four mutant residues (N67S, V87D, R136H and D386N) in the extracellular domain (ECD) were found in clinical samples from breast cancer patients. We developed a mutant TrkB (mtTrkB)-fused mCherry expression plasmid, which enabled expression of mutant TrkB-fused mCherry from a single lentivirus plasmid (Fig. 5A). We used yeast display and a cell–cell interaction format to select antibodies against the stable 293 T cell line overexpressing mtTrkB, which was tagged with C-terminal mCherry fusion. The yeast display construct expressed both antibodies and intracellular EGFP via a T2A sequence. Therefore, we can sort the yeast–mammalian cell complexes based on dual fluorescent (EGFP and mCherry) to isolate antibodies against mtTrkB. A stable cell line of wild-type TrkB (wtTrkB) fused with mCherry was also generated to serve as a negative control. The screening utilized four rounds of selection. For each round, we used fluorescence-activated cell sorting (FACS) to enrich yeast cells that selectively bound to the cell line expressing mutant TrkB (Fig. 5B). As expected, variants with higher affinity for mtTrkB were enriched throughout the selection process. The post-selection yeast antibody libraries were sequenced, and the selected antibodies were expressed to confirm their binding to mtTrkB cell lines. These studies showed that four rounds of selection were sufficient to enrich for antibodies against the mutant TrkB protein (Fig. 5C).

### Validation of anti-mtTrkB antibodies

**Figure 6:**
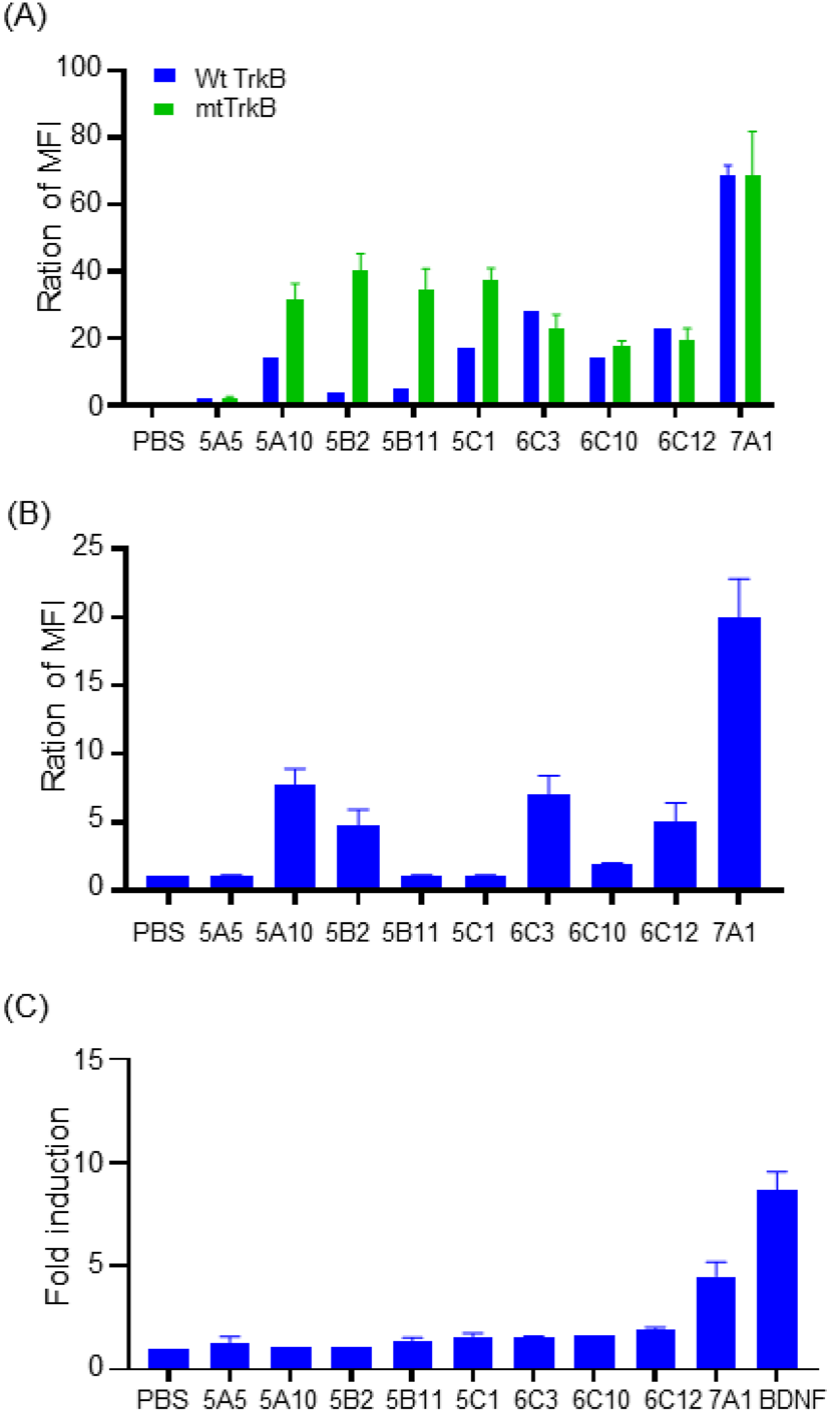
Validation of anti-mutated TrkB antibodies. (**A**) Comparison of cell binding with anti TrkB mutation antibodies (10 μg/mL) in wide-type TrkB (wtTrkB), and TrkB mutation (mtTrkB, N67S, V87D, R136H and D386N) stable cell lines by flow cytometry. (**B**) Determination of cell binding in breast cancer cell line MDA-MB-231 with anti-mtTrkB antibodies by flow cytometry. (**C**) TrkB-CRE-bla 293 reporter cell lines were treated with BDNF (100 ng/mL), anti TrkB mutation antibodies at 10 μg/mL or isotype antibody for 5 h. Cells were incubated with the CCF4-AM substrate and subjected to FACS based on the FRET signal. All data were repeated and normalized to the PBS control group. Abbreviation: Mean fluorescence intensity (MFI).

We isolated antibodies that selectively bind the mutant form of TrkB using the cell–cell interaction format described above. Four rounds of selection were carried out to enrich for antibodies that selectively bound mtTrkB-fused mCherry overexpressing cells. Ninety-six clones were sequenced to analyze the enrichment. Some sequences appeared in multiple clones, and these were selected for more in-depth study. We compared the binding of these anti-mtTrkB antibodies (10 μg/mL) with stable cell lines of wtTrkB and mtTrkB by flow cytometry. The results showed some antibodies (6C12, 7A1) recognized both mutant and wild type TrkB, but two (5B2 and 5B11) only recognized the mutant form of TrkB (Fig. 6A). Next, to determine whether these antibodies bind wtTrkB on the surface of cancer cells, we used MDA-MB-231, a triple negative breast cancer cell line expressing wtTrkB, for further binding studies. Antibodies 5B11 did not bind to wtTrkB when presented on the surface of this breast cancer cell line. (Fig. 6B). In addition, we also tested whether these antibodies are agonists. The TrkB-CRE-bla 293 reporter cell line was treated with Brain-derived neurotrophic factor (BDNF) at 100 ng/mL (positive control) or anti-mtTrkB antibody at 10 μg/mL for 5 h. Cells were incubated with the CCF4-AM substrate, and subjected to FACS analysis based on the FRET signal. Unlike the agonist effect of anti-wtTrkB antibodies seen in our previous work, these antibodies to mtTrkB showed no significant agonist activity (Fig. 6C) indicate they did not trigger downstream signaling of TrkB. Taken together, these results showed that the 5B11 antibody only recognizes the mutant form of TrkB. This platform of cell–cell interaction can be used to select antibodies against mutant TrkB.

## Discussion

Approximately 20% of breast cancers are “triple negative”, meaning they do not express any of the cell surface receptors (estrogen, progesterone, and Her2) commonly found in breast cancer. However, they have been shown to express TrkB at a level significantly elevated compared to normal tissue^34^. TNBCs are associated with a poor outcome, not only because they are aggressive in nature, but also because they lack easily identifiable cell surface proteins appropriate for the development of targeted immunotherapy. In order to address the urgent need for improved TNBC therapies, we focused on ADCs, and two key challenges in their development. The first is identifying a cell surface antigen which is selectively expressed on tumors and allows for efficient internalization of a payload to kill the cancer cells. Identifying an appropriate target is a critical parameter that effects the efficacy of any form of immunotherapy. The second challenge is to identify the optimum coupling method for conjugation of the specific antibody and payload to achieve the desired efficacy profile and suitability for large scale manufacturing. There are many examples of ADCs that showed early preclinical promise, but did not progress and were terminated due to safety or efficacy concerns. Here, we used a screening platform to identify antibodies specifically recognizing mutant receptors in order to reduce targeting of normal cells. Of course, for clinical use, more extensive in vitro and in vivo testing of our ADC construct needs to be done to further validate its safety and efficacy profiles. For example, tissue cross-reactivity tests using various human biological specimens are needed to verify the specificity of the antibody. Also, animal experiments using MDA-MB231, MCF-7 or patient-derived xenografts, and eventually human trials are needed to determine if TrKB-ADCs can be developed as an effective therapy. In addition, a stable linkage between antibody and cytotoxic agent is a key aspect of ADC. A stable ADC linker ensures that less of the cytotoxic load is shed before reaching tumor cells, improves safety, and limits dose. We need to further investigate the safety, pharmacokinetic, pharmacodynamic and dosing of ADCs to make them safer when used in the clinical setting.

A main purpose of this work was to address the first challenge by shedding light on an under-appreciated growth factor receptor, TrkB, that is expressed on the surface of breast cancer cells, including TNBCs. Unlike its more famous counterpart, Her2, it has yet to be used as a target for immunotherapies, including ADC and CAR-T cells. TrkB expression has been shown to be associated with a wide range of cancers. TrkB activation can trigger a cascade of signals, including the phosphatidylinositol 3-kinase (PI3K), Ras–mitogen-activated protein kinase (MAPK), and the PLCγ pathway. Activation of these signaling pathways induces oncogenic effects by increasing cancer cell growth, proliferation, survival, migration and epithelial-to- mesenchymal transition^35–39^. In addition, it is reported that BDNF-TrkB signaling can regulate cancer-endothelial cell interaction and affect the recurrence and metastasis of TNBC^40,41^. These findings have important therapeutic significance because they provide one of the directions of TrkB targeted therapy for breast cancer patients. In the current study we show that TrkB is expressed across HER2+ and HER2− breast cancer cell lines, providing an incentive to investigate its suitability as an ADC target. As is the case with many other immunotherapy targets, TrkB is expressed at low basal levels in non-cancerous healthy human tissues and at higher levels in tumors^42–44^. However, based on our prior studies^16^ showing TrkB is immunogenic in some breast cancer patients we theorized that TrkB may be an especially good target for immunotherapy. TrkB’s increased immunogenicity in the cancer setting suggests some form of mutation occurs so that it is sufficiently different chemically to terminate tolerance. This would allow us to use modern immunochemical techniques to develop targeted therapies with an even great therapeutic index, since normal and cancer cells would differ not only in the level of expression, but also in the chemical nature of the target.

We built an anti-TrkB DVD-ADC, then tested it against breast cancer cell lines. The upper variable domain of the DVD is targeting TrkB present on cancer cells, and the lower variable domain is a catalytic antibody that is used for site-specific conjugation of a cytotoxic payload. Lys99 is the only site for conjugation with β-lactam functionalized MMAF. The integrated catalytic antibody provides an easy and rapid method for drug conjugation. After conjugation, the anti-TrkB DVD-ADC had lost its catalytic activity, suggesting quantitative conjugation to Lys99. Since the DVD-ADC is composed of two heavy and light chains, the drug-to-antibody ratio (DAR) is 2. The anti-TrkB DVD-ADC was cytotoxic for TrkB-expressing breast cancer cell lines, including HER2− TNBC cell lines that were unaffected by a corresponding anti-HER2 DVD-ADC.

In summary, we herein report construction of what we believe to be the first ADCs targeting TrkB, and show that our anti-TrkB DVD-ADC armed with MMAF is cytotoxic for multiple breast cancer cell lines, including three TNBC lines. In addition, we describe a cell–cell interaction format which we used to successfully select antibodies which bind to mutant forms of TrkB associated with breast cancer, but not to wild type TrkB. If the initial promise of these experiments is confirmed in the clinical setting, we would suggest the following protocol: First, screen the sera of patients with breast cancer to identify patients making antibodies to TrkB, (or for that matter, to any other growth factor receptor). Second, in cases where such antibodies are identified, use sequencing studies to determine if TrkB expressed on the cell surface of the patient’s cancer is mutated. Third, if mutated, use modern immunochemical technologies to isolate antibodies that selectively bind to the mutant form on cancer cells, and do not bind to the normal protein. Fourth, use these selective antibodies to construct ADC or Cart-T cells for tumor therapy. We feel that such a protocol would not be limited to breast cancer, and could be applicable to many other solid tumors.

## Materials and methods

### Establishment of cell lines expressing wtTrkB and mtTrkB

The mutated residues of NTRK2 (TrkB gene) were analyzed using cBioPorta (http://cbioportal.org/), which contains multidimensional cancer genomics and clinical data from TCGA. Four mutant residues (N67S, V87D, R136H and D386N) in the ECD were found in the samples from breast cancer patients and used for mutated TrkB. The wtTrkB or mtTrkB fusion mCherry genes were synthesized by IDT and cloned into Lentivirus expression vectors. The lentivirus plasmid was transfected into 293 T cells along with packaging plasmids for the production of lentivirus. 293 T cells were transduced with the lentivirus to integrate wtTrkB or mtTrkB into the genome. The wtTrkB and mtTrkB stable cell lines with high mCherry expression were sorted by FACS.

### Yeast display screening with mammalian cells

A yeast antibody library was generated and screened as described previously^45^. For each round of enrichment, yeast cells were cultured in 50 ml SDCAA (2.0% glucose, 0.67% yeast nitrogen base, 0.5% casamino acids, 0.54% disodium phosphate and 0.86% monosodium phosphate) for 24 h at 28 °C with shaking at 250 r.p.m. The final OD600 was ∼1.7. Yeast cells were centrifuged at 4 °C and 4,500 r.p.m. for 20 min, then resuspended in 50 ml SGCAA (2% Galactose, 0.67% yeast nitrogen base, 0.5% casamino acids, 0.54% disodium phosphate and 0.86% monosodium phosphate), with an OD600 of ∼0.5 for induction. Yeast cells were cultured at 20 °C with shaking at 250 r.p.m. to reach an OD600 of 1.5. 5 ml of the yeast culture was centrifuged, washed twice with PBS, and resuspended in PBS. For yeast screening, cells expressing wtTrkB without mCherry were first incubated with the yeast library to eliminate binding of yeast to wtTrkB. The yeast library, now minus any cells binding to wtTrkB, was then co-incubated the mtTrkB-mCherry cells at a ratio of 1:10. The mixture was incubated for an hour at room temperature, then washed with FACS buffer and analyzed with FACS. Positive cell–cell complexes expressing both GFP and mCherry were identified and collected in SDCAA media.

### Sequencing of recovered antibody genes in yeast cells and antibody expression

Plasmids were extracted from at least 10^7^ yeast cells per sample using Zymoprep Yeast Plasmid Miniprep II (Zymo Research, Irvine, CA) according to the manufacturer’s instructions. E. coli were transformed with yeast plasmids and single colonies were sequenced with primer 5’-GAG GCT CTG CAC AAC CAC TAC ACG. For further assay, scFv-Fc fusion proteins were generated. The yeast display vectors were digested with SfiI enzyme and the scFv cDNA was subcloned into a pFUSE expression vector (pfusehg1fc; Invivogen), which contains the Fc domain of human IgG1.

The expression vectors were transfected into Expi293F cells using ExpiFectamine 293 reagent, and the supernatant was collected 5 days after transfection. A HiTrap Protein A HP column (no. 17-0403-03; GE Healthcare) was used to affinity-capture antibodies using the ÄKTA purifier 100 (GE Healthcare). Antibodies were then eluted with glycine buffer (pH 2.7). Eluted antibody was neutralized with Tris buffer and stored in PBS after buffer exchange using Ultracel 30 kDa (Merck Millipore). The concentration of antibodies was determined by Qubit Protein Assay Kit (Thermo Fisher Scientific).

### Cell binding assay by flow cytometry

Breast cancer lines (5 × 10^5^ cells) were stained with 1 μg anti-TrkB mAb^15^ or isotype control in 100 μL FACS buffer (PBS solution with 1% FBS) on ice. After washing with FACS buffer, the cells were incubated with anti-human Fc-conjugate PE (Invitrogen, 12-4998-82) for 30 min. After washing twice with 200 µL ice-cold FACS buffer, the cells were analyzed using a Flow Cytometer LSRII (BD Biosciences). Data were analyzed using FlowJo.

### TrkB reporter cell assay

TrkB-CRE-bla 293 reporter cell lines^16^ were treated with BDNF or anti mtTrkB antibodies for 5–6 h. The adherent cells were loaded with CCF4-AM dye, treated with Accutase detachment solution, and subjected to FACS. Upon activation, the reporter cells expressed beta-lactamase, cleaved CCF4, and disrupted the FRET; excitation at 405 nm produced a florescent signal at 450 nm, while the green florescent signal at 520 nm was diminished.

### DVD engineering

Anti-TrkB DVD-encoding plasmids were constructed by linking the VH and VL of mAb 641^16^ to the VH and VL of h38C2^46^ via a short hexapeptide linker (ASTKGP). The desired DNA sequences were synthesized as gBlocks (Integrated DNA Technologies) and expressed with a human IgG1 heavy chain or κ light chain constant domain. The DVD expression cassettes were cloned into mammalian expression vector pcDNA3.1 and transiently transfected into Freestyle 293 cells (Thermo Fisher Scientific). The supernatants were collected at 5-day intervals, followed by filtration and purification using 5-mL HiTrap Protein G HP columns (GE Healthcare) in conjunction with an ÄKTA FPLC instrument (GE Healthcare) and HiLoad 16/600 Superdex 200 pg column in size exclusion chromatography (ÄKTA pure). The purity of DVD was confirmed by SDS-PAGE followed by Coomassie staining, and the concentration was determined by measuring the absorbance at 280 nm. The expression and purification of the anti-HER2 DVD was previously described^46^.

### pHAb dye conjugation

Anti-TrkB mAb 641 was conjugated with pHAb dye (Promega) using amine chemistry according to the manufacturer recommended protocol. Briefly, for conjugating the pHAb dye to surface Lys residues, the antibody was reacted with an excess of amine reactive pHAb dye (5–20 molar excess) for 1 h. Free dye was subsequently removed using a Zeba desalting column (Thermo Fisher Scientific).

### Antibody internalization

Breast cancer cell lines were used to study internalization of pHAb dye-conjugated anti-TrkB mAb 641. Cells were plated at 5 × 10^4^ cells per well overnight, then 10 µg/mL antibody-pHAb dye conjugate was added to the cells at 37 °C for various time periods (1–16 h). Following this incubation, the cells were immediately analyzed by flow cytometry.

### DVD-ADC conjugation

The β-lactam-MMAF was synthesized as described^21^. A previously published protocol was used for DVD-ADC assembly^47^. Briefly, all conjugations were performed in PBS (pH 7.4) after the antibodies were concentrated to 50 µM (10 mg/L) using a 30-kDa cutoff centrifugal filter device (Millipore). Next, 6 µL of β-lactam-MMAF (1 mM in 10% (v/v) DMSO in PBS; 10 eq) was added to 300 µg of antibody for a final reaction volume of 36 µL. The solution was incubated for 4 h at room temperature. All conjugations were deemed complete by loss of catalytic activity using a catalytic activity assay (described below) for which a portion of the crude reaction diluted to 1 µM in PBS was used. Upon completion, unreacted compound was removed by using a PD-10 desalting column (GE Healthcare). The conjugates in PBS were stored at 4 °C for short term use and at − 80 °C in aliquots for long term use. The concentration was determined by measuring the absorbance at 280 nm.

### Catalytic activity assay

Catalytic activity was analyzed using methodol as described^47^. DVDs or IgG1s were diluted to 1 µM in PBS (pH 7.4) and dispensed in 98-µL aliquots into a 96-well plate in triplicate. Then, 2 µL of 10 mM methodol in ethanol was added and the fluorescence was assessed immediately using a SpectraMax M5 instrument (Molecular Devices) with SoftMax Pro software, a wavelength of excitation (λ ext) set to 330 nm, a wavelength of emission (λ em) set to 452 nm, and starting at 0 min using 5-min time intervals. The signal was determined by normalizing to 98 µL PBS with 2 µL of the methodol solution added.

### In vitro cytotoxicity assay

SKBR3, MDA-MB-468 and MDA-MB-231 breast cancer cells were seeded at a density of 5 × 10^3^ cells/well in 96-well plates. After 24 h culture, cells were exposed to unconjugated antibody and ADCs at 37 °C for 72 h. After incubation for 72 h, the cell viability was measured using the CellTiter 96 AQueous One Solution Cell Proliferation Assay (Promega) following the manufacturer’s instructions. The cell viability was calculated as a percentage of untreated cells (defined as 100%). Sample sizes, statistical tests, and definitions of error bars are indicated in the figure legends and were calculated using GraphPad Prism 8. Differences with *P < 0.05 were considered statistically significant.

## Supplementary Information

Supplementary Information.
