# Supplementary material for: A new immunochemical strategy for triple-negative breast cancer therapy
Source: Sci Rep. 2021 Jul 21;11:14875. doi: 10.1038/s41598-021-94230-4 (PMC8295383; doi:10.1038/s41598-021-94230-4)
Supplement: Supplementary file 1 — Supplementary Information. [file 41598_2021_94230_MOESM1_ESM.docx]

**A new immunochemical strategy for triple negative breast cancer therapy**

Chih-Wei Lin, Tianqing Zheng, Geramie Grande, Alex R. Nanna, Christoph Rader, Richard A. Lerner^1^

**Affiliations:**

^a^ Department of Chemistry, The Scripps Research Institute, La Jolla, CA 92037

^b^ Department of Immunology and Microbiology, The Scripps Research Institute, Jupiter, FL 33458, USA.

^1^ Correspondence to rlerner@scripps.edu

This file includes:

Supplementary Fig. S1-S2


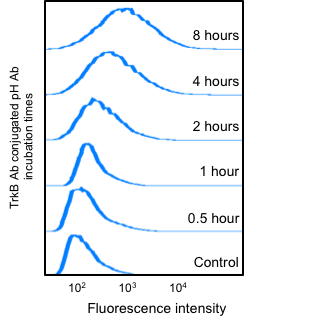


**(B)**


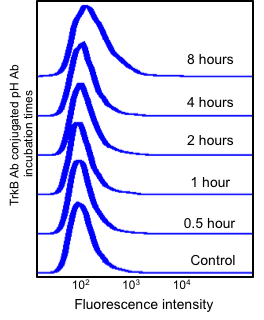


**Supplementary Fig. S1. Internalization of anti-TrkB mAb 641 by breast cancer cell lines.** Flow cytometry analysis of 10 µg/mL TrkB-pHAb dye internalizing at 37°C into (A)MDA-MB-468 or (B) MDA-MB-231 cells at various timepoints. The increase in fluorescence intensity over time indicates internalization.

**
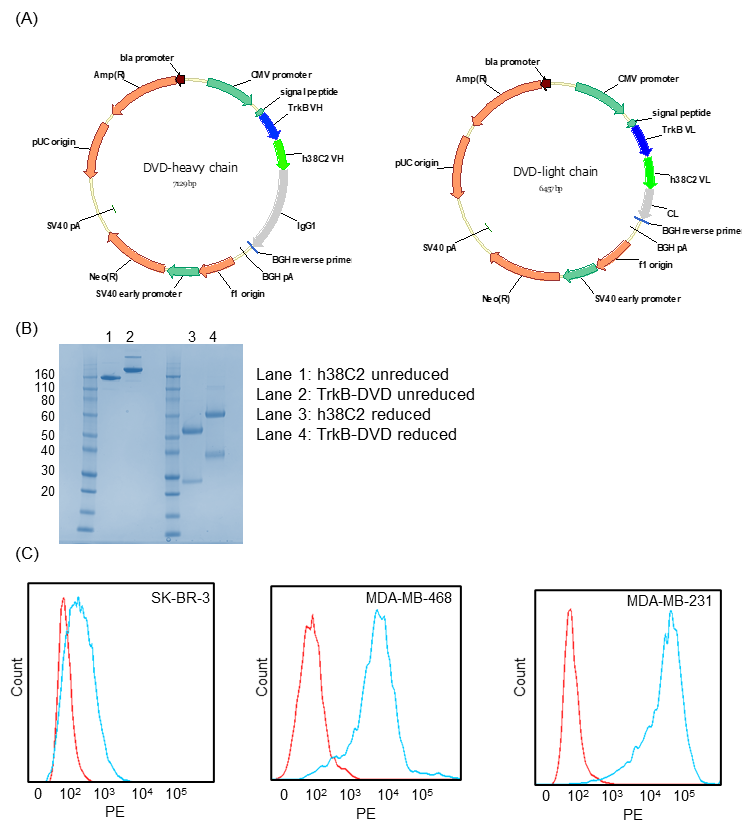
**

**Supplementary Fig. S2. Construction and characterization of anti-TrkB DVD.** (A) Map of anti-TrkB DVD-encoding plasmids. The DVD expression cassettes were cloned into mammalian expression vector pcDNA3.1 via *BamHI* and *PmeI*. (B) Analysis of purified anti-TrkB DVD by SDS-PAGE and Coomassie blue staining. (C) Flow cytometric analysis of breast cancer cell lines (5x10^5^ cell) using anti-TrkB DVD (blue) or isotype control antibody (red) with a secondary anti-human-PE (blue) antibody
